# A Retrospective Comparison of Hemodynamic and Clinical Outcomes between Two Differently Designed Aortic Bioprostheses for Small Aortic Annuli

**DOI:** 10.3390/jcm10051063

**Published:** 2021-03-04

**Authors:** Do Jung Kim, Sak Lee, Hyun-Chel Joo, Young-Nam Youn, Kyung-Jong Yoo, Seung Hyun Lee

**Affiliations:** 1 Department of Thoracic and Cardiovascular Surgery, Ajou University Hospital, Ajou University School of Medicine, 164 World cup-ro, Yeongtong-gu, Suwon 16499, Korea; DJkim119@aumc.ac.kr; 2 Division of Cardiovascular Surgery, Department of Thoracic and Cardiovascular Surgery, Severance Cardiovascular Hospital, College of Medicine, Yonsei University, 50-1 Yonsei-ro, Seodaemun-gu, Seoul 03722, Korea; SAK911@yuhs.ac (S.L.); VIETCOMM@yuhs.ac (H.-C.J.); YNYOUN@yuhs.ac (Y.-N.Y.); KJY@yuhs.ac (K.-J.Y.)

**Keywords:** heart valve prosthesis implantation, hemodynamic monitoring, prosthesis design, small aortic annulus

## Abstract

The Trifecta valve has externally mounted leaflets; it differs from classic internally mounted valves (e.g., Carpentier-Edwards [C-E]). We evaluated post-implantation hemodynamics and clinical outcomes of these bioprostheses in small aortic annuli. From January 2015 to April 2019, 490 patients who underwent aortic valve replacement (AVR) were reviewed retrospectively. Altogether, 183 patients received 19 or 21 mm diameter C-E (*n* = 121) or Trifecta (*n* = 62) prostheses. To minimize confounding variables, we performed propensity-score matching analysis. The mean transvalvular pressure gradient (TVPG) was significantly lower in the Trifecta than in the C-E group at discharge (12.9 ± 4.8 vs. 15.0 ± 5.3 mmHg, *p* = 0.044). TVPG change over time was not significantly different between groups (*p* = 0.357). Left ventricular mass index decreased postoperatively (reduction: C-E, 28.1%; Trifecta, 30.1%, *p* = 0.879). No late mortality, severe patient–prosthesis mismatch, moderate-to-severe paravalvular leakage, structural valve degeneration, or valve thromboses were observed. Freedom from valve-related events at 3 years were similar for C-E (97.9% ± 2.1%) and Trifecta (97.7% ± 2.2%) patients (log-rank *p* = 0.993). Bioprosthesis design for small annuli significantly affected TVPG immediately after AVR. However, hemodynamics over time and clinical outcomes did not differ between the two designs.

## 1. Introduction

With the growth in the elderly population, bioprosthetic valves are being increasingly used in patients with symptomatic aortic valve disease. Bioprosthetic valves have low thrombogenic risk and do not require continuous use of anticoagulants. Although bioprosthetic valves have improved in durability and excellent hemodynamic performance [1], patient–prosthesis mismatch (PPM) is a potential issue in patients undergoing aortic valve replacement (AVR), particularly in patients with a small annulus [2]. To minimize PPM, which is associated with worse clinical outcomes and decreased survival [3], the effective orifice area (EOA) of the implanted prosthesis should be maximized. Supra-annular aortic bioprostheses, such as the Carpentier-Edwards (C-E) PERIMOUNT Magna valves (Edwards Lifesciences Corp, Irvine, CA, USA) and Trifecta valves (St. Jude Medical, Inc., St. Paul, MN, USA), were introduced to counteract this issue, and both valves have optimized EOA and low transvalvular gradients [4,5,6].

The Trifecta valve has a different design from that of the classic internally mounted C-E valve, with externally mounted leaflets to maximize the valve opening. Despite these significant differences, studies comparing post-implantation hemodynamic and clinical outcomes of these two valves are limited [6,7], and in particular, few studies have compared these outcomes among Asian patients with a small aortic annulus [2]. Therefore, the aims of this study were to evaluate the early hemodynamic performance of these two differently designed valves and to review the clinical outcomes of small-sized bioprostheses in patients undergoing AVR.

## 2. Materials and Methods

### 2.1. Study Population

Between January 2015 and April 2019, 490 consecutive patients who had undergone AVR with a bioprosthesis were retrospectively reviewed at Severance Cardiovascular Hospital, Yonsei University College of Medicine. Patients who had undergone sutureless aortic valve implantation (*n* = 121) and received other bioprostheses (*n* = 32) or homografts (*n* = 3) were excluded from the analysis. In addition, patients who had undergone an aortic root enlargement procedure (*n* = 7), potentially affecting the hemodynamics and clinical results, were excluded from the study. Among the remaining 327 patients, 183 patients with a small aortic annulus had received an AVR with a 19 mm or 21 mm diameter bioprostheses (C-E: *n* = 121 and Trifecta: *n* = 62) and were enrolled in this study.

The study procedures were performed according to the guidelines stipulated in the Declaration of Helsinki, and the study was approved by the Institutional Review Board of Yonsei University College of Medicine (approval number: 4-2018-0274). The need to obtain individual informed patient consent was waived because of the retrospective nature of the study.

### 2.2. Surgical Technique

AVR with a bioprosthesis was performed using median or partial upper sternotomy with standard cardiopulmonary bypass and mild hypothermia (32 °C). Antegrade cold blood cardioplegia provided myocardial protection. After excision of the diseased aortic valve and decalcification of the annulus, the bioprosthesis type was selected according to the surgeon’s preference. The appropriate bioprosthetic valve size was determined by measuring the aortic annulus using a standard manufacturer’s sizer. The bioprosthesis was implanted in the supra-annular position using non-everting mattress sutures. Postoperatively, all patients received oral warfarin as anticoagulation treatment for the first 3 months, with a target international normalized ratio (INR) of 2.5. However, patients who also used antiplatelet agents received anticoagulation treatments based on an INR of 2.0 (range: 1.8–2.2) to reduce the risk of bleeding.

### 2.3. Echocardiographic Assessment

Echocardiographic parameters, including left ventricular (LV) ejection fraction, peak and mean transvalvular pressure gradients (TVPG), aortic valve area (AVA) or EOA, and left ventricular mass index (LVMI), were measured according to established guidelines [8]. LV ejection fraction was assessed using the Simpson method, and the transvalvular gradients were derived from the transaortic flow using the simplified Bernoulli equation with continuous-wave Doppler [8]. LV mass was calculated using the Devereux formula [9] and normalized to body surface area (BSA) to obtain LVMI. The AVA or EOA was calculated from the continuity equation by using the LV outflow tract area and the time velocity integral. At discharge, the indexed EOA (IEOA) was calculated as EOA (information on the type and size of each bioprosthesis was obtained from the manufacturers) divided by the patient’s BSA. PPM was classified as moderate (0.65 < IEOA ≤ 0.85 cm^2^/m^2^) or severe (IEOA ≤ 0.65 cm^2^/m^2^) [10].

### 2.4. Data Collection and Outcomes

Follow-up clinical information was obtained from the patients’ medical records or through telephonic interview. We obtained data on cause of death, provided by Statistics Korea, a central organization for statistics under the Ministry of Strategy and Finance, for the study period. The clinical mean follow-up duration was 2.5 ± 1.2 years. Patients underwent transthoracic echocardiography by two independent experienced cardiologists preoperatively at discharge, 1 year after surgery, and biennially thereafter if available. All patients (100%) underwent an echocardiogram at discharge. Follow-up echocardiography was available in 87.3% and 67.1% of the patients at 1 and 3 years, respectively. However, assessment of in vivo EOA by echocardiogram was available in 97.9%, 85.1%, and 66.3% of the propensity-score matched patients at each of the above time points. The echocardiographic follow-up duration was 1.6 ± 0.6 years in the C-E group and 2.1 ± 0.8 years in the Trifecta group. The study follow-up was closed in October 2019.

The primary endpoint was the hemodynamic performance (change in TVPG and LVMI) of the bioprostheses at discharge and during the follow-up period. The secondary endpoints were early and late adverse events (classified as events occurring ≤30 and >30 days after AVR, respectively), overall survival, and valve-related events at the follow-up time points. Early outcomes were defined using the Society of Thoracic Surgeons’ guidelines [11]. Operative mortality was defined as any death within 30 days after surgery, or during the same hospital admission. “Valve-related events” were defined as a composite outcome of structural valve deterioration (SVD), pacemaker implantation, endocarditis, valve thrombosis, severe PPM, and valve-related mortality. SVD was defined as an increase in the mean aortic gradient by > 20 mmHg, with a concomitant decrease in EOA by >0.6 cm^2^ (and/or a decrease in the Doppler velocity index > 0.15) during follow-up, leading to severe aortic stenosis, and/or new onset or increase of intraprosthetic regurgitation causing moderate or severe aortic regurgitation [12]. Prosthesis paravalvular regurgitation, valve thrombosis, and endocarditis were not considered SVDs.

### 2.5. Statistical Analysis

Statistical analyses were performed using R software version 3.6.1 (available through R-project online). Continuous variables are presented as the mean ± standard deviation when normally distributed or as the median [interquartile range] when non-normally distributed. Categorical variables are presented as absolute numbers and percentages. Groups were compared using a Student’s t-test or Mann–Whitney U test for continuous variables, and a chi-square test or Fisher’s exact test for categorical variables, as appropriate.

To correct for potential confounders between the groups, we performed propensity-score matching analyses. Propensity scores were derived using a separate logistic regression model that included patients’ demographical characteristics (i.e., age, gender, body surface area, hypertension, diabetes, and chronic renal failure) and operative data (isolated AVR and concomitant procedures) as shown in Table 1. Valve size, aortic cross-clamp (ACC) time, and cardiopulmonary bypass (CPB) time were not included in the propensity model. Propensity scores were then matched to obtain pairs of matched patients. In this study, we adopted widely used matching methods, such as optimal matching. Discrimination and calibration of propensity scores were assessed with C statistics and Hosmer–Lemeshow statistics (*p* = 0.829 and *p* = 0.736), respectively. The balance of covariates between groups was assessed by standardized mean differences with adequacy considered as < 0.2. Comparisons within the matched cohort were performed using a paired-sample t-test or Wilcoxon signed-rank test for continuous variables, and a McNemar’s test for categorical variables. After matching, mixed-effects models were used to further investigate the changes in echocardiographic measurements over time and to account for the correlation between repeated follow-up measurements according to the bioprosthesis type. Piecewise-linear models, using time as a continuous measure and a random effect parameter for the patients, were constructed with knot values at times A (preoperative) and B (from discharge up to 3 years postoperative). Interaction of valve type (group: C-E vs. Trifecta) and postoperative time course of TVPG and LVMI were assessed. Overall survival and freedom from valve-related events were compared using the Kaplan–Meier estimator for survival analysis and the log-rank test. Statistical significance was defined as a 2-sided *p*-value < 0.05.

## 3. Results

### 3.1. Patient Characteristics and Operative Results

The baseline characteristics of the patients are reported in Table 1. Before matching, the C-E group had a higher prevalence of previous percutaneous coronary intervention, a higher EuroScore II, and fewer bicuspid-valve pathologies than the Trifecta group (all *p* < 0.05). Moreover, the C-E group tended to have a higher prevalence of atrial fibrillation (AF; *p* = 0.052) and rheumatic pathology than the Trifecta group (*p* = 0.054). After the application of matching, all variables were appropriately balanced and were similar in both groups (*n* = 47 in each).

The 19 mm bioprosthesis was more frequently used in the Trifecta group than in the C-E group (before matching: 27.4% vs. 18.2%, *p* = 0.149 and after matching: 27.7% vs. 12.8%, *p* = 0.072), although this differential preference of usage was not significant between the two groups. After risk adjustment, the distribution of surgeries, such as isolated AVR and AVR with concomitant procedures (multiple valvular surgery and ascending aorta replacement), was similar in both groups (Table 1).

### 3.2. Early and Late Clinical Outcomes

**Figure 1:**
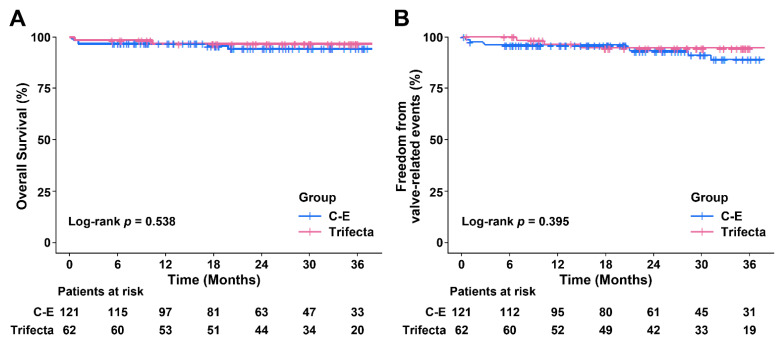
Survival and freedom from valve-related events in the unmatched cohort: (**A**) The Kaplan–Meier estimate of survival for the Carpentier-Edwards (C-E, blue line) and Trifecta (red line) valves; (**B**) The same Kaplan–Meier estimate of freedom from valve-related events.

In the whole cohort, there were 5 cases of in-hospital mortality caused by bowel perforation, pneumonia, and heart failure in the C-E group, and by brain hemorrhage in the Trifecta group. The actuarial survival at 3 years was 94.3% ± 2.3% and 96.6% ± 2.4% (log-rank *p* = 0.538) in the C-E and Trifecta groups, respectively (Figure 1A).

There was no difference in early postoperative complications between the two groups (Table 2). During the follow-up period, 1 patient in the Trifecta group experienced prosthetic valve endocarditis, which required reoperation, at 7 months postoperatively. In the C-E group alone, 3 patients (2.5%) underwent pacemaker implantation and 5 patients (4.1%) had severe PPM. There were no patients with SVD in either group, and there was no reoperation due to SVD. Only 1 patient in the C-E group developed subclinical (possible) SVD (increase in mean TVPG of > 10 mm Hg with concomitant decrease in EOA >0.3 cm^2^), compared with discharge echocardiographic assessment. Moreover, both groups had no abnormal changes in aortic valve morphology (thickening, calcification, flail leaflets, or pannus), impaired mobility of bioprosthetic valve leaflets, or disruptions of any valvular components (strut/frame). Valve-related events were observed in 9 (7.4%) and 3 (4.8%) patients receiving C-E and Trifecta, respectively. The actuarial freedom from valve-related events at 3 years was 88.9% ± 3.8% and 94.5% ± 3.1% in the C-E and Trifecta groups, respectively (log-rank *p* = 0.395; Figure 1B), and it was not significantly related to the valve type.

After matching, in-hospital mortality (2.1% vs. 2.1%; *p* >0.999) was similar in both groups, and there was no late mortality. Early clinical outcomes were not different between the two groups (Table 2). None of the patients experienced moderate-to-severe paravalvular leakage, severe PPM, valve thrombosis, or SVD at follow-up. The incidence of moderate PPM did not differ significantly between the two groups (C-E, 12.8% vs. Trifecta, 14.9%; *p* = 0.765). In the Trifecta group, infective endocarditis was diagnosed in the same patient as before the matching, and reoperation was required. Valve-related events occurred in 1 (2.1%) patient each from the C-E and Trifecta groups. However, time-related events (mortality and valve-related events) for the matched cohort hardly occurred; therefore, the Kaplan–Meier curve could not be used.

### 3.3. Hemodynamic Results for Matched Patients

**Figure 2:**
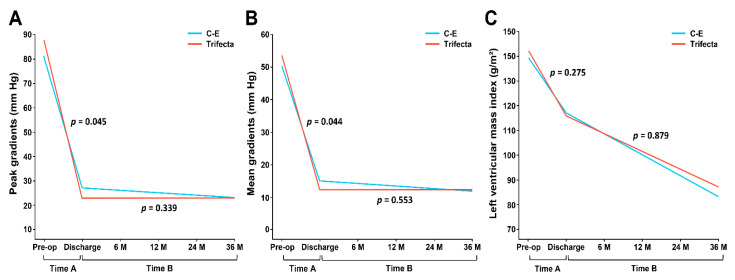
Changes in echocardiographic measurements over time for the Carpentier-Edwards (C-E) and Trifecta valves: (**A**) Peak gradient from baseline to discharge (Time A) and from immediately after surgery to 3 years postoperatively (Time B); (**B**) Mean gradients following the same timeline; (**C**) Left ventricle mass index (LVMI). No significant time-valve effects on gradients or LVMI were found. *p*-values shown for analyses from baseline to discharge (Time A) and the interaction of the mixed model.

As shown in Table 3, peak and mean TVPGs at discharge were significantly lower in the Trifecta group than in the C-E group (24.0 ± 9.0 vs. 27.9 ± 10.4 mm Hg, *p* = 0.045; 12.9 ± 4.8 vs. 15.0 ± 5.3 mm Hg, *p* = 0.044, respectively). However, at 1 and 3 years postoperatively, there were no significant differences between the two groups. For the interaction of valve type and postoperative time course (Time B in Figure 2), there was no significant effect in either the peak (*p* = 0.339) or mean (*p* = 0.553) TVPGs. This is followed by a sustained and stable pattern of TVPG throughout the postoperative period of 3 years in both groups (Figure 2A,B).

The Trifecta group had significantly larger theoretical IEOAs (calculated by dividing the manufacturer’s EOA by the BSA) at discharge than that of the C-E group (1.01 ± 0.10 vs. 0.96 ± 0.08 cm^2^/m^2^, *p* = 0.001), but the in vivo IEOAs (EOA measured by echocardiogram) were similar in both groups (*p* = 0.995). In addition, LVMI in the C-E and Trifecta groups decreased from 137.5 ± 40.1 g/m^2^ and 145.3 ± 39.0 g/m^2^ at baseline to 98.9 ± 20.7 g/m^2^ and 101.6 ± 24.1 g/m^2^, at 3 years postoperatively, respectively (reduction rate: 28.1% and 30.1%). As shown in Figure 2C, although LVMI decreased steadily over time after surgery, no differences were noted between the two groups (*p* = 0.879).

**Figure 3:**
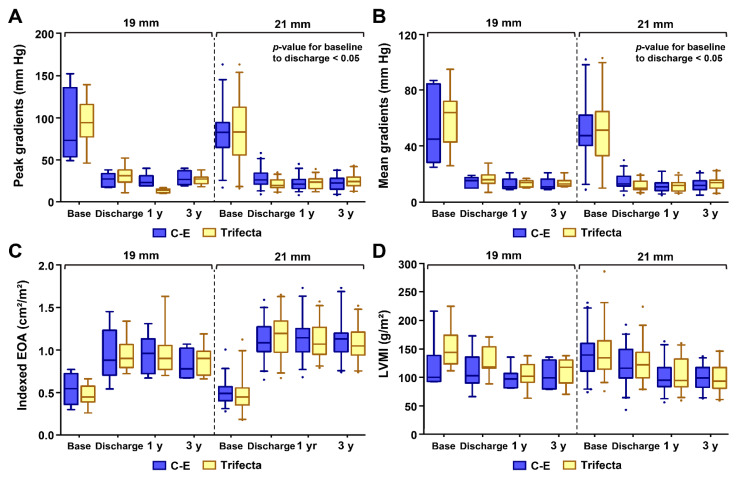
Distribution of echocardiographic values for each prosthetic size: (**A**) The distribution of the peak gradient; (**B**) Mean gradient distribution; (**C**) Indexed effective orifice area (IEOA) distribution; and (**D**) Left ventricular mass index (LVMI) distribution, for both prosthetic sizes (19 mm and 21 mm) in the matched patients; *p*-values of the peak and mean transvalvular pressure gradients from baseline to discharge alone were statistically significant in patients that received the 21 mm diameter valve (*p*-value < 0.05).

Similar echocardiographic findings were found when the data were stratified by valve size for the C-E and Trifecta bioprostheses (Figure 3). *p*-values of the peak and mean TVPG from baseline to discharge alone were statistically significant in patients that received the 21 mm diameter valve.

## 4. Discussion

In the matched patients with a small aortic annulus, the Trifecta group had significantly lower TVPG and larger theoretical IEOA at discharge than the C-E group. However, for up to 3 years postoperatively, there was no significant difference in TVPG between the two groups. LVMI in both groups showed a substantial and steady decrease over the study period compared to the preoperative period, with no significant time-related changes found between the two groups. Furthermore, no severe PPM, significant paravalvular leakage, or valve thrombosis was identified in either groups. Before and after matching, overall survival, and valve-related events were not significantly related to valve type.

**Figure 4:**
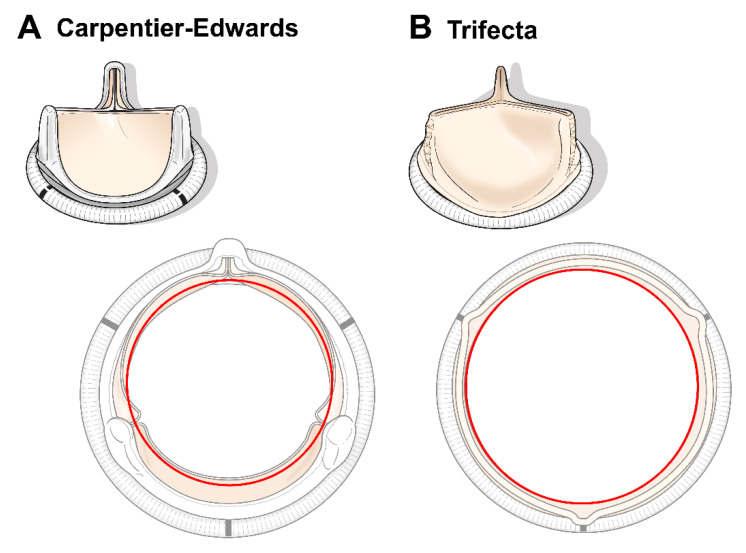
Two different valve designs: (**A**) Carpentier-Edwards (C-E) valve with internally mounted leaflets; (**B**) Trifecta valve with externally mounted leaflets. The Trifecta valves are associated with a larger effective orifice area than the C-E valves.

AVR for the small aortic annulus requires implantation of a small-sized prosthesis, which could lead to a high incidence of PPM, procedural complications, and residual TVPG. To ensure placement of the most appropriately sized valve and to optimize the hemodynamic results, surgeons may consider several strategies, including an AVR with aortic root enlargement, implantation of a rapid deployment (or sutureless) valve, use of a supra-annular valve, and different suture techniques. We focused on two bioprostheses, which differed according to the presence of internal or external mounting of leaflets around the strut. The C-E valve, with three pericardial leaflets, is internally mounted under a flexible stent, whereas the Trifecta valve is an externally mounted valve with a single sheet of pericardial tissue wrapped around a titanium stent [5]. Theoretically, the leaflets of the Trifecta valve are able to open fully. This prevents reduction of blood flow and ensures a relatively larger internal diameter, which should result in a low TVPG and a large EOA (Figure 4). It was confirmed that the 19 mm and 21 mm diameter Trifecta valves had EOA values (provided by the manufacturers) of at least 0.1 cm^2^ larger than those of the C-E valves.

Previous studies have shown that the Trifecta valve is beneficial in terms of achieving lower pressure gradients and higher EOA immediately after surgery [6,7,13]. However, Wendt et al. reported no correlations between these values and valve type in a multivariate covariance analysis [6]. Tadokoro et al. showed that the early hemodynamic advantage of the Trifecta valve lasted for up to 1 year postoperatively. Thereafter, this difference diminished gradually over time [14]. These results are partially consistent with our findings, showing that the differences in the TVPG between the two groups did not change over time, with the only exception being the time immediately after surgery. Therefore, despite performing a propensity-score matching analysis, we will need to consider patient-related factors that could affect the hemodynamic performance, such as concomitant surgery and preoperative characteristics, as it is difficult to explain these clinical and hemodynamic outcomes by prosthetic design alone. Furthermore, the surgeon’s surgical experience and the suture technique used can also affect TVPG and EOA [15].

Generally, LV mass regresses by 20–30% after AVR [16,17,18]. In our study, both groups demonstrated continuous reduction in LVMI from after surgery until the last follow-up, as compared to that before surgery (C-E: 28.1% reduction; Trifecta: 30.1% reduction). As previously reported, a marked reduction in LV afterload following an AVR considerably decreases LV systolic and diastolic pressures, thereby reducing mean LV pressure [18,19]. In a recent study published by Rubens et al., they reported that the Trifecta valve had a significantly greater LV mass regression than the C-E valve (45.5 g/m^2^ vs. 28.3 g/m^2^, *p* = 0.016),and improved mid-term clinical composite outcomes of readmission, congestive heart failure, and all-cause mortality [20]. Moreover, Une et al. [21] showed that LV mass regression in both patients with aortic stenosis (AS) and those with aortic regurgitation (AR) occurred at a steep decline over 2 years after AVR. Vollema et al. [22] reported that LV mass regression and changes in LV global longitudinal strain were similar despite different preoperative LV remodeling in patients with AS and those with AR. These findings are consistent with our results. After matching, 15–17% of patients with AR were included in the LV mass regression analysis; however, LVMI steadily decreased in both valves regardless of the pathophysiology of AS and AR (from baseline to 3 year follow-up; AS, 139.2 ± 35.7 g/m^2^ to 100.6 ± 21.8 g/m^2^; AR, 152.7 ± 55.5 g/m^2^ to 99.2 ± 26.4 g/m^2^, respectively). In our study, although it was not possible to prove the effect of valve design on LVMI, each valve showed significantly effective hemodynamic performance in terms of LVMI and PPM.

It is well known that PPM after AVR increases the TVPG, causes left ventricular hypertrophy, or interferes with LV mass regression, and these effects may increase long-term mortality and lead to a poor prognosis [19,23]. However, in the current study, none of the patients had severe PPM, and the rate of moderate PPM was 12.8% and 14.9% for the C-E and Trifecta groups, respectively. Interestingly, our study was performed on Asian patients only, who tend to have a lower BSA compared to western patients. These acceptable PPM outcomes for both valves may be due to smaller BSAs, even when EOA is similar (our mean BSA: 1.54 m^2^, compared to 1.8–1.95 m^2^ in other studies [7,13,24]).

Moreover, some studies have reported that PPM and increasing postoperative pressure gradients are associated with SVD [25,26]. Flameng et al. [26] demonstrated that small valve size, anti-calcification treatment, and PPM were independent predictors of SVD. Other factors, including younger age, male sex, and porcine valve, also promoted SVD [27,28]. In fact, SVD refers to morphological abnormalities of valve leaflets associated with hemodynamic dysfunction in our study. Although the follow-up period was short, there was no occurrence of clinically relevant SVD, and only 1 patient who received a C-E valve in the unmatched cohort had subclinical SVD. Subclinical changes in hemodynamic valve function may not be noticeable, but these findings have great importance to clinicians. The rate of SVD is < 10% at 10 years after surgical AVR; however, its incidence progressively increases thereafter. In a study of the C-E PERIMOUNT valve in the aortic position by Forcillo et al. [29], they reported an actuarial freedom from reoperation for prosthetic valve dysfunction at 10 and 20 years, which were 96% ± 1% and 67% ± 4%, respectively. Johnstone et al. [25], in their report of 12,569 patients, demonstrated that actuarial estimates of explant due to SVD at 10 and 20 years were 1.9% and 15%. With the Trifecta aortic bioprosthesis, the 6-year freedom from SVD and reoperation are 95% and 96%, respectively [30]. However, some reports of early Trifecta failure caused by cusp tears, early excessive pannus formation in the inflow portion, and leaflet calcification in the outflow portion have been described [31,32]. A study by Stubeda et al. [33] showed that Trifecta aortic valves were associated with a significantly high risk of early reoperation in patients aged < 60 years and in patients that were current smokers. Given that the risk of early reoperation for SVD due to multiple factors other than mechanical issue could gradually increase, careful echocardiogram surveillance may be warranted in patients with high postoperative TVPG and severe PPM.

Over time, reoperation in patients with bioprostheses will be required because of SVD, and the valve-in valve transcatheter AVR is a viable option for selected patients with high surgical risk. Coronary artery ostial obstruction during valve-in-valve implantation is a potentially devastating complication that could cause myocardial ischemia. The externally mounted leaflets of the Trifecta valves may increase the risk of coronary obstruction. Furthermore, the titanium struts and rigid sewing cuff of the Trifecta may not allow its fracture to facilitate valve-in-valve implantation. Therefore, to optimize the outcomes of future valve-in-vale procedure in patients with Trifecta failure, the type and size of the bioprosthesis used should be carefully selected during AVR surgery.

This study had several limitations. First, it was a non-randomized, retrospective study performed in a single center; thus, a selection bias influencing the decision in bioprosthetic valve choice may have existed. Given that the number of patients with a Trifecta valve was relatively small, and the preoperative characteristics and operative procedures in both groups were significantly different, we used a propensity-score matching analysis to minimize statistical error. Furthermore, in our institution, Perimount Magna ease valves were introduced in late 2018, and Trifecta-GT is rarely used. Therefore, patients with the most recent valves were excluded from this study. Second, this study had a short follow-up period and a relatively small sample size, which made it difficult to make statistically robust inferences. Nevertheless, the study has provided us with useful information by focusing on Asian patients with smaller body sizes, on whom AVR was performed using 19 mm or 21 mm small-sized valves. Therefore, further large-scale prospective studies are necessary to determine the effects of two differently designed valves in patients with small aortic annuli. Third, good hemodynamics at rest may have been overestimated because during exercise, the TVPGs may increase and PPM may become more evident. Furthermore, missing values for the echocardiography results may have biased our findings regarding valve hemodynamics. Especially, hemodynamic performance over time should be interpreted with caution because only two-thirds of the patients underwent echocardiography at 3 years after surgery, causing the data available for analysis to be limited. Finally, we compared the two groups using the in vitro EOA provided by manufacturer values at discharge because the patients’ hemodynamic states, such as cardiac output and ventricular function, were not stabilized immediately after surgery, and a poor acoustic window made the measurement of EOA by echocardiogram difficult. However, for the mixed effect model analysis to reflect the physiological hemodynamic flow in actual patients, subsequent echocardiographic data based on in vivo EOA was used, and those data were obtained for 97.9%, 85.1%, and 66.3% of living patients at discharge, 1 year, and 3 years after surgery, respectively. Although echocardiography was not performed in all patients, we believe that the results of this study were meaningful because data from a substantial number of patients with echocardiogram data were used. Furthermore, given the effectiveness of EOA in the prediction of PPM, a sufficient number of patients with echocardiogram data should be examined to predict long-term outcomes.

## 5. Conclusions

The Trifecta valve demonstrated improved IEOA and low TVPG at discharge, compared to the C-E valve, but no difference in terms of TVPG was found during the follow-up period. Both valves also resulted in sufficient LV reverse remodeling and favorable clinical outcomes. Although the two differently designed valves did not affect the hemodynamics and clinical outcomes of the patients with small annuli, further large-scale studies are needed to confirm long-term clinical outcomes.

## Figures and Tables

**Table 1:** Baseline characteristics and operative data before and after propensity score matching analysis.

Variables ^a^	Unmatched			Matched		
	C-E (*n* = 121)	Trifecta (*n* = 62)	*SMD*	C-E (*n* = 47)	Trifecta (*n* = 47)	*SMD*
Patient Demographics						
Age (years)	73.3 ± 6.5	72.0 ± 7.9	0.180	73.5 ± 5.6	73.4 ± 4.3	0.021
Female	100 (82.6)	47 (75.8)	0.169	37 (78.7)	38 (80.9)	0.053
Body surface area (m^2^)	1.54 ± 0.14	1.56 ± 0.14	0.113	1.55 ± 0.12	1.55 ± 0.12	0.025
Hypertension	82 (67.8)	38 (61.3)	0.156	31 (66.0)	33 (70.2)	0.108
Diabetes mellitus	38 (31.4)	15 (24.2)	0.161	15 (31.9)	13 (27.7)	0.093
Chronic renal failure	19 (15.7)	5 (8.1)	0.238	2 (4.3)	3 (6.4)	0.095
Cerebrovascular accidents	25 (20.7)	7 (11.3)	0.258	5 (10.6)	5 (10.6)	<0.001
Chronic lung disease	10 (8.3)	5 (8.1)	0.007	4 (8.5)	3 (6.4)	0.081
Peripheral artery disease	17 (14.0)	4 (6.5)	0.127	3 (6.4)	3 (6.4)	<0.001
Coronary artery disease	34 (28.1)	14 (22.6)	0.393	10 (21.3)	10 (21.3)	0.121
Previous PCI	17 (14.0)	2 (3.2)	0.252	2 (4.3)	1 (2.1)	<0.001
Previous cardiac surgery	14 (11.6)	4 (6.5)	0.179	1 (2.1)	2 (4.3)	0.121
Atrial fibrillation/flutter	40 (33.1)	12 (19.4)	0.315	10 (21.3)	10 (21.3)	<0.001
Logistic EuroSCORE (%)	15.7 ± 16.5	12.8 ± 14.7	0.186	12.6 ± 12.2	12.4 ± 14.8	0.011
EuroSCORE II (%)	7.7 ± 12.1	4.9 ± 7.9	0.278	4.7 ± 5.3	4.8 ± 8.3	0.014
NYHA III-IV	63 (52.1)	29 (46.8)	0.106	21 (44.7)	23 (48.9)	0.094
Valve pathology						
Degenerative	60 (49.6)	33 (53.2)	0.073	25 (53.2)	27 (57.4)	0.086
Bicuspid	13 (10.7)	15 (24.2)	0.360	11 (23.4)	9 (19.1)	0.104
Rheumatic	28 (23.1)	7 (11.3)	0.318	8 (17.0)	6 (12.8)	0.120
Endocarditis	10 (8.3)	4 (6.5)	0.069	2 (4.3)	2 (4.3)	<0.001
Prosthetic failure	8 (6.6)	2 (3.2)	0.157	1 (2.1)	2 (4.3)	0.121
Aortic stenosis	78 (64.5)	41 (66.1)	0.035	32 (68.1)	32 (68.1)	<0.001
Aortic regurgitation	16 (13.2)	12 (19.4)	0.167	7 (14.9)	8 (17.0)	0.058
Mixed aortic lesion	15 (12.4)	7 (11.3)	0.034	7 (14.9)	5 (10.6)	0.128
Operative data						
Valve size ^b^			0.292			0.529
19 mm	22 (18.2)	17 (27.4)		6 (12.8)	13 (27.7)	
21 mm	99 (81.8)	45 (72.6)		41 (87.2)	34 (72.3)	
Isolated AVR	38 (31.4)	32 (51.6)	0.419	22 (46.8)	25 (53.2)	0.110
Concomitant procedures						
Coronary artery bypass	13 (10.7)	8 (12.9)	0.067	7 (14.9)	6 (12.8)	0.062
Mitral valve surgery	54 (44.6)	8 (12.9)	0.748	9 (19.1)	8 (17.0)	0.055
Tricuspid valve surgery	37 (30.6)	5 (8.1)	0.595	6 (12.8)	5 (10.6)	0.066
Aorta replacement	8 (6.6)	10 (16.1)	0.303	7 (14.9)	6 (12.8)	0.062
Surgical ablation	16 (13.2)	5 (8.1)	0.168	4 (8.5)	5 (10.6)	0.072
ACC time (min) ^b^	93.1 ± 40.4	79.0 ± 27.6	0.385	81.4 ± 37.3	80.5 ± 28.9	0.027
CPB time (min) ^b^	120.2 ± 48.2	108.7 ± 36.8	0.257	103.8 ± 41.9	110.6 ± 38.0	0.170

^a^ Values are presented as mean ± standard deviation or *n* (%). ^b^ not included in the propensity model. ACC = aortic cross-clamp; AVR = aortic valve replacement; C-E = Carpentier-Edwards; CPB = cardiopulmonary bypass; EuroSCORE = European System for Cardiac Operative Risk Evaluation; NYHA = New York Heart Association; PCI = percutaneous coronary intervention; SMD = standardized mean differences.

**Table 2:** Early and late clinical outcomes of the unmatched and matched patients

Variables ^a^	Unmatched			Matched		
	C-E (*n* = 121)	Trifecta (*n* = 62)	*p*-Value	C-E (*n* = 47)	Trifecta (*n* = 47)	*p*-Value
Early results						
Reoperation for bleeding	8 (6.6)	3 (4.8)	0.752	1 (2.1)	1 (2.1)	>0.999
Renal failure	5 (4.1)	2 (3.2)	>0.999	1 (2.1)	1 (2.1)	>0.999
Prolonged ventilation	9 (7.4)	4 (6.5)	>0.999	2 (4.3)	3 (6.4)	>0.999
Cerebrovascular events	4 (3.3)	2 (3.2)	>0.999	1 (2.1)	2 (4.3)	>0.999
Atrial fibrillation	20 (16.5)	11 (17.7)	0.836	9 (19.1)	8 (17.0)	0.789
Operative mortality	4 (3.3)	1 (1.6)	0.664	1 (2.1)	1 (2.1)	>0.999
Late results						
Infective endocarditis	0 (0)	1 (1.6)	0.339	0 (0)	1 (2.1)	>0.999
Pacemaker implantation	3 (2.5)	0 (0)	0.552	1 (2.1)	0 (0)	>0.999
Paravalvular leakage						
Mild	3 (2.5)	1 (1.6)	>0.999	1 (2.1)	1 (2.1)	>0.999
Moderate–severe	0 (0)	0 (0)		0 (0)	0 (0)	-
Patient–prosthesis mismatch						
Moderate	22 (18.2)	11 (17.7)	0.942	6 (12.8)	7 (14.9)	0.765
Severe	5 (4.1)	0 (0)	0.169	0 (0)	0 (0)	-

^a^ Values are presented as *n* (%). C-E = Carpentier-Edwards; SVD = structural valve deterioration.

**Table 3:** Echocardiographic results of the matched patients at each time point.

Variables ^a^	Group	Baseline	Discharge	1-Year	3-Year	*p*-Value *	*p*-Value ^†^
Peak gradient (mmHg)	C-E	82.5 ± 32.8	27.9 ± 10.4	22.8 ± 8.3	22.8 ± 8.1	0.045	0.339
	Trifecta	87.6 ± 34.2	24.0 ± 9.0	23.1 ± 6.7	25.5 ± 7.1		
Mean gradient (mmHg)	C-E	50.9 ± 22.2	15.0 ± 5.3	11.9 ± 4.4	12.5 ± 4.5	0.044	0.553
	Trifecta	53.9 ± 23.4	12.9 ± 4.8	12.2 ± 3.6	13.7 ± 3.9		
Aortic valve area (cm^2^)	C-E	0.79 ± 0.24	1.71 ± 0.35	1.74 ± 0.35	1.66 ± 0.34	0.895	0.112
	Trifecta	0.73 ± 0.26	1.70 ± 0.36	1.65 ± 0.30	1.56 ± 0.29		
Indexed EOA (cm^2^/m^2^) ^b^	C-E	0.51 ± 0.15	1.10 ± 0.24	1.12 ± 0.23	1.08 ± 0.24	0.995	0.097
	Trifecta	0.47 ± 0.18	1.11 ± 0.25	1.07 ± 0.22	1.02 ± 0.21		
LVMI (g/m^2^)	C-E	137.5 ± 40.1	119.0 ± 34.5	100.8 ± 24.5	98.9 ± 20.7	0.275	0.879
	Trifecta	145.3 ± 39.0	126.5 ± 31.5	103.5 ± 26.5	101.6 ± 24.1		

^a^ Values are presented as mean ± standard deviation. ^b^ The indexed EOA was calculated by dividing the in vivo EOA, measured using echocardiogram, by the patient’s body surface area; it was evaluated in 97.9% (*n* = 92/94), 85.1% (*n* = 80/94), and 66.3% (*n* = 61/92) of the matched patients at each time point. * *p*-value: Baseline vs. discharge. ^†^ *p*-values for change from discharge to 3 years postoperatively are based on the estimates from the mixed model. C-E = Carpentier–Edwards; EOA = effective orifice area; LVMI = left ventricular mass index.

## Data Availability

The data presented in this study are available on request from the corresponding author. The data are not publicly available due to privacy.
